# Identification of FOXP3^+^ epithelial cells contributing to pancreatic proliferation and angiogenesis

**DOI:** 10.1152/ajpcell.00461.2023

**Published:** 2023-12-04

**Authors:** Ruining Gong, Xianghan Chen, Xiaoyuan Sun, Yuxing Zhang, Jia Wang, Qian Yu, Ke Lei, He Ren

**Affiliations:** ^1^Shandong Provincial Key Laboratory of Clinical Research for Pancreatic Diseases, Center for GI Cancer Diagnosis and Treatment, Tumor Immunology and Cytotherapy, Medical Research Center, The Affiliated Hospital of Qingdao University, Qingdao, People’s Republic of China; ^2^Department of Gastroenterology, The Affiliated Hospital of Qingdao University, Qingdao, People’s Republic of China

**Keywords:** angiogenesis, epithelial, FOXP3, proliferation, regulatory T cell

## Abstract

Forkhead box protein 3 (FOXP3), traditionally recognized as a specific transcription factor for regulatory T cells (Tregs), has also been identified in various tumor epithelial cells (named as cancer-FOXP3, c-FOXP3). However, the natural state and functional role of FOXP3 positive tumor epithelial cells remain unknown. Monoclonal cells expressing varying levels of c-FOXP3 were isolated from established PANC-1 cells using limited dilution. Whole transcriptome sequencing and weighted gene co-expression network analysis (WGCNA) were conducted on these subsets, followed by in vitro and in vivo functional investigations. In addition, we identified c-FOXP3^+^E-cadherin^−^ epithelial cells in human pancreatic cancer tissues after radical resection by immunofluorescence co-staining. We also investigated the connection between c-FOXP3^+^E-cadherin^−^ epithelial cells and their clinicopathological features. Our study uncovered a distinct subset of c-FOXP3^+^ tumor epithelial cells characterized by reduced E-cadherin expression. C-FOXP3^+^E-cadherin^−^ cells displayed significant proliferation potential and pro-angiogenic effect through the expression of chemokines, including C-X-C motif ligand 1 (CXCL1), C-X-C motif ligand 5 (CXCL5), and C-X-C motif ligand 8 (CXCL8). Notably, higher counts of c-FOXP3^+^E-Cadherin^−^ cells correlated with poorer prognosis, lower tumor differentiation, lymph node metastasis, and vascular invasion in pancreatic ductal adenocarcinoma (PDAC). In conclusion, this work revealed the stable expression of FOXP3 in tumor epithelial cells, marking a distinct subset. C-FOXP3^+^E-cadherin^−^ epithelial cells exhibit active proliferation and promote angiogenesis in a vascular endothelial growth factor A (VEGFA) independent manner. These findings provide novel insights into PDAC prognosis and therapeutic avenues.

**NEW & NOTEWORTHY** In this study, we revealed a novel c-FOXP3^+^ tumor epithelial cell subset marked by diminished E-cadherin and stable FOXP3 expression. These subpopulations not only show robust proliferation and drive angiogenesis via CXCL1, CXCL5, and CXCL8, bypassing VEGFA pathways, but their heightened presence also correlates with adverse PDAC outcomes. By challenging traditional epithelial cell definitions and extending lymphocyte markers to these cells, our findings present innovative targets for PDAC treatment and enrich our understanding of cell biology.

## Graphical abstract

**Figure:**
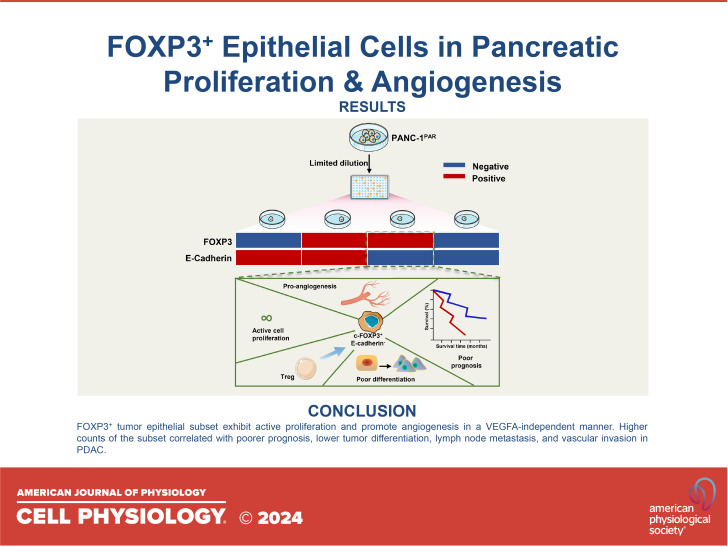

## INTRODUCTION

The forkhead box P3 (FOXP3) is a member of the forkhead/winged helix family of transcription factors, which plays an important role in the development and function of CD4^+^CD25^+^ T regulatory cells (Tregs) (1–3). FOXP3 had been recognized as a specific intracellular regulatory marker for Tregs. FOXP3^+^Tregs play a crucial role in maintaining immune balance and are vital suppressors of anti-tumor immune responses (4, 5).

Recent evidence demonstrates that FOXP3 expression is not confined to Tregs, and it is also identified in epithelial cells. Various tumor types, including pancreatic, breast, ovarian, prostate, lung, and melanoma, have reported the FOXP3 expression, referred to as cancer-FOXP3 (c-FOXP3) (6–12). The role of c-FOXP3 remains controversial, as it appears to function as a tumor activator or a suppressor in different tumor cells (9, 13–16).

Previous studies have predominantly focused on the roles of the c-FOXP3 molecule in tumor cells. However, the functions of naturally occurring c-FOXP3^+^ tumor epithelial cells remain unclear. This has prompted us to investigate the characteristics of c-FOXP3^+^ tumor epithelial cells. As a nuclear transcription factor, FOXP3 is challenging to be a marker for cell enrichment. Previously, we demonstrated that established pancreatic ductal adenocarcinoma (PDAC) cell lines, such as PANC-1, BxPC-3, and AsPC-1 expressed c-FOXP3 at different levels (12, 16). PANC-1, widely used in pancreatic cancer research, is known for its mixed-like cell morphology(17–19). We obtained c-FOXP3^+^ tumor epithelial cells in their natural state by using a limited dilution method with PANC-1.

In this study, we isolated and cultured c-FOXP3^+^ tumor epithelial cells successfully, and explored their molecular expression, functional characteristics, and clinical relevance. Our research unveils the stabilization of a functional subset of c-FOXP3^+^ tumor epithelial cells within PDAC for the first time, and expands the theoretical implications of transcriptional regulation involving lymphocyte markers within tumor epithelial cells.

## MATERIALS AND METHODS

### Human Samples

A total of 75 PDAC samples were collected from patients who had radical surgery at the Affiliated Hospital of Qingdao University (Qingdao, China) in 2016. All patients who did not undergo chemotherapy and radiotherapy before surgery were included, mean age of 61.8 ± 9.3 yr (range 36–79), 45 male and 30 female. The use of these specimens and patient information was approved by the Ethics Committee of the Affiliated Hospital of Qingdao University (Permission No. QYFYWZLL28017). All patients gave written consent, as per the ethics committee’s guidelines, allowing the use of their specimens and information for subsequent research.

### Cell Culture

Human pancreatic adenocarcinoma PANC-1 cells (CLS Cat. No. 300228/p635_Panc-1, RRID: CVCL_0480) were purchased from the American Type Culture Collection (ATCC, Manassas, VA). To acquire highly purified FOXP3 expression cells, parental PANC-1 cells (PANC-1^PAR^) were seeded in 96-well plates by using limited dilution. After a single cell developed into a single clone, the cells were isolated and cultured in Dulbecco’s Modified Eagle Media (DMEM, PM150210; Procell Life Science & Technology Co., Ltd, Wuhan, China) containing 10% fetal bovine serum (FBS, 164210-50; Procell Life Science & Technology Co., Ltd, Wuhan, China) and 1% penicillin/streptomycin (P/S, PB180120; Procell Life Science & Technology Co., Ltd, Wuhan, China) in a 37°C incubator with 5% CO_2_ and a humidified atmosphere.

### Quantitative Real-Time Polymerase Chain Reaction

The total RNA was extracted from PANC-1^PAR^ and four single cell-derived cells using the Total RNA purification kit (AG21101; Accurate Biotechnology, Changsha, China), and cDNA was synthesized using One Scrip cDNA synthesis kit (AG11711; Accurate Biotechnology, Changsha, China) as directed by the manufacturer. The quantitative real-time polymerase chain reaction (qPCR) was performed with SYBR Green *Pro Taq* HS Premix (AG11701; Accurate Biotechnology, Changsha, China) and the CFX96 Real-Time System (Bio-Rad, CA). *GAPDH* served as an internal control, and the mRNA levels of target genes were calculated using the 2^-ΔΔCt^ method. *hFOXP3* primer: forward CACAACATGCGACCCCCTTTCACC and reverse AGGTTGTGGCGGATGGCGTTCTTC. *hE-Cadherin* primer: forward CAGTCTAGGCCAGTGCATCA and reverse TTGCCCTCTGCTTTGTTCTT. *hCXCL1* primer: forward GAAAGCTTGCCTCAATCCTG and reverse CTTCCTCCTCCCTTCTGGTC. *hCXCL5* primer: forward AGCTGCGTTGCGTTTGTTTAC and reverse TGGCGAACACTTGCAGATTAC. *hCXCL8* primer: forward ACTGAGAGTGATTGAGAGTGGAC and reverse AACCCTCTGCACCCAGTTTTC. *hVEGFA* primer: forward AGGAGTACCCTGATGAGATCGAGTA and reverse TGGTGAGGTTTGATCCGCATA. All qPCR primer sequences were obtained from BGI TECH SOLUTIONS (BEIJING LIUHE) CO., LIMITED.

### Western Blotting

In lysis solution (R0010; Solarbio, Beijing, China) containing protease inhibitors (HY-K0010; MedChemExpress, Monmouth Junction, NJ), PANC-1^PAR^ and four single cell-derived cells lysates were produced. The lysates were centrifuged at 12,000 rpm at 4°C for 15 min, and the supernatant was collected. Protein quantification was performed using the BCA protein concentration determination kit (PC0020; Solarbio, Beijing, China). The protein samples were prepared with 5X SDS-PAGE loading buffer (P0015L; Beyotime, Shanghai, China) and heated at 100°C in a metal bath for 10 min. Approximately 30 μg of total protein was loaded into each well of 10% SDS-PAGE gels. Electrophoresis was carried out, and proteins were transferred onto 0.45 μm PVDF membrane (Merck Millipore, Boston, MA). After blocking at room temperature for 1 h, the membranes were incubated with the primary antibody at 4°C for 14–16 h. The primary antibodies used were as follows: FOXP3 (1: 1,000; ab191416; Abcam, Cambridge, MA, UK), E-cadherin (1:500; ab76055; Abcam, Cambridge, MA, UK), GAPDH (1:5,000; 60004-1-Ig; Proteintech Group, Inc., Chicago). Following primary antibody incubation, the membranes were washed with phosphate-buffered saline containing 1% Tween-20 (PBST) and incubated with the secondary antibody (A0208, A0216; Beyotime, 1:5,000, Shanghai, China) at room temperature for 1 h. Subsequently, the signal was detected by chemiluminescent substrate (WBKLS0500; Merck Millipore, Boston, MA) and Alliance Q9 chemiluminescence imaging system (UVITEC, Cambridge, UK).

### EdU Assay

The logarithmic growth phase cells were planted at a density of 4 × 10^3^ per well in 96-well plates. Prepare 50 μM EdU (C10310-1; RIBOBIO Biological, Guangzhou, China) media by diluting EdU solution with cell culture medium 1,000:1 and incubating it for 2 h. The cells were fixed with 4% paraformaldehyde (P0099; Beyotime, Shanghai, China), treated with a staining reaction solution for 30 min, and then immediately viewed under a microscope (×20, Leica, Wetzlar, Germany).

### Subcutaneously Implanted Tumor Model

Six-week-old male BALB/c Nude mice were purchased from Beijing Vital River Laboratory Animal Technology Co., Ltd. [License No: SCXK (Beijing) 2021-0011]. Mice were housed in an SPF environment at the Medical Research Center, The Affiliated Hospital of Qingdao University. PANC-1^PAR^ and four monoclonal cells (1 × 10^7^/mL cells) were suspended in a 1:1 mixture of phosphate-buffered saline (PBS, PB180327; Procell, Wuhan, China) and Matrigel (354234; Corning Inc., Corning, NY). PANC-1^PAR^ and four monoclonal cells (200 μL per mouse, *n* = 6 per group) were implanted in the axilla of the mice’s right forelimb. The size of the tumors was measured with a vernier caliper every 4 days. Tumor volumes = (length × (width)^2^/2). The mice were euthanized when the tumor reached 1 cm^3^. The Committee for the Care and Use of Laboratory Animals at the Affiliated Hospital of Qingdao University granted permission under No.: AHQU-MAL20201016.

### Tube Formation Assays

For the preparation of conditioned medium (CM) of PANC-1^PAR^ and monoclonal cells and then used to suspend the human umbilical vein endothelial cells (HUVECs) (CC-Y1285; EK-Bioscience, Shanghai, China) to 1 × 10^5^/mL. A 96-well plate was coated with growth factor reduced Matrigel (354234; Corning Inc., Corning, NY), and 100 μL HUVEC suspension was dispensed into each well of the coated 96-well plate. The plates were incubated at 37°C with 5% CO_2_ for 4 h and stained with Calcein AM (C2012; Beyotime, Shanghai, China). Fluorescence microscopy (Leica, Wetzlar, Germany) was used for visualization. Vessel density was calculated by averaging the tube numbers in five randomly selected fields at ×10 magnification per well.

### Immunohistochemistry and Fluorescent Co-staining

Immunohistochemical (IHC) staining of mouse subcutaneous tumors and human PDAC paraffin tissue samples was performed following standard procedures. Briefly, all sections were deparaffinized with xylene and rehydrated with gradual ethanol. High-pressure antigen retrieval was conducted using the appropriate antigen retrieval buffer in accordance with the antibody manufacturer’s instructions. Each section was blocked with 10% goat serum blocking solution (ZLI-9021; Beijing, China) for 30 min at 37°C. The primary antibody was applied and incubated overnight at 4°C. The primary antibodies used were as follows: FOXP3 (1:200; ab191416; Abcam, Cambridge, MA, UK), E-Cadherin (1:500; ab76055; Abcam, Cambridge, MA, UK), Ki-67 (1:1,200; GB111499; Servicebio, Wuhan, China) and CD31 (1:2,000; ab182981; Abcam, Cambridge, MA, UK). A universal mouse/rabbit secondary antibody (PV-9000; Beijing, China) was incubated at 37°C for 30 min. DAB (ZLI-9018; Beijing, China) staining was performed, followed by counterstaining with hematoxylin (G1140; Solarbio, Beijing, China). After slide dehydration, the sections were mounted for observation under a microscope (Leica, Wetzlar, Germany).

Co-staining was performed using four-color multiple fluorescent immunohistochemical staining kit (pika universal secondary antibody) (abs50012; Absinbio, Shanghai, China). The steps of co-staining were performed, according to the manufacturer’s protocol. The primary antibodies/fluorescent dyes used were: anti-E-Cadherin (1:500; ab76055; Abcam, Cambridge, MA, UK)/TSA 570 (1:100), anti-FOXP3 (1:200; ab191416; Abcam, Cambridge, MA, UK)/TSA 520 (1:100). Sections were treated with rabbit/mouse horseradish peroxidase-conjugated (HRP) secondary antibody (#M505D01; Absinbio, Shanghai, China) for 15 min at 37°C. DAPI working solution (1×) was added to cover the sample area and incubated at room temperature for 5 min. Images were obtained using Phenolmager Fusion (Akoya Biosciences, Inc., A Delaware Corporation).

### F-actin Staining

After 24 h of cell climbing, the cells were washed twice with PBS and fixed with 3.7% formaldehyde in PBS. Dilute Actin-Tracker Red-Rhodamine (C2207S; Beyotime, Shanghai, China) at a ratio of 1:100, add 200 µL staining working solution to each slide, and incubate at room temperature for 30 min in the dark. After the dropwise addition of DAPI Staining Solution (C1005; Beyotime, Shanghai, China), the slides were mounted with an anti-fluorescence quencher (P0126; Beyotime, Shanghai, China) and viewed using a Confocal Microscope (Leica, Wetzlar, Germany).

### Co-expression Networks Construction

Various gene networks were developed and modularized using the WGCNA R program at various stages. Hierarchical clustering and dynamic tree-cutting function detection were employed by the modules. To link modules with function features, module membership (MM) and gene significance (GS) were assessed. The modules with the highest Pearson module membership correlation (MM) and a *P* absolute value of 0.05 were designated as hub modules.

### Statistical Analysis

The software GraphPad Prism9.0 (RRID: SCR_002798; San Diego, CA) was used for data analysis. Each experiment was independently repeated at least three times. The data were presented as means ± standard deviation (SD). Data of Ki-67 index and CD31 count in subcutaneous tumor are presented as the median. The significance of the difference between means was determined by one-way ANOVA followed by Duncan’s test or Tukey’s test. The log-rank Mantel–Cox test was used to evaluate the significance of the differences observed in Kaplan–Meier curves. Categorical data were evaluated by the Pearson’s chi-square test. *P < 0.05, **P < 0.01, ***P < 0.001, ****P < 0.0001.

## RESULTS

### Subclones Derived from PANC-1 Cells Exhibit Diversity of C-FOXP3 Expression Pattern and Cell Morphology

**Figure 1.**
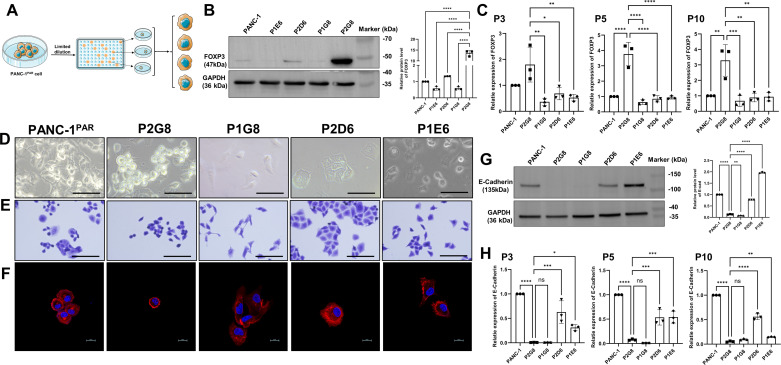
FOXP3^+^ epithelial cells were isolated in vitro. *A*: diagram of the experiment. PANC-1 cell suspension was subjected to cell counting and dilution using a limited dilution method. The cells were seeded into 96-well plates. The different single-cloned cells were expanded and cultured, followed by subsequent molecular validation and functional studies. *B*: FOXP3 protein levels in PANC-1^PAR^ and monoclonals lysates were examined by western blot. Intensities of immunoblots were quantified by ImageJ software (Image Processing and Analysis in Java; National Institutes of Health; http://imagej.nih.gov/); (*n* = 3). *C*: FOXP3 mRNA levels were measured at the passage of 3, 5, and 10 in PANC-1^PAR^ and monoclonals by qPCR; (*n* = 3). *D*: contrast images of cells under 2-D culture conditions (×40, scale bar = 100 μm). *E*: crystalline violet staining in PANC-1^PAR^ and monoclonal cells (×40, scale bar = 100 μm). *F*: F-actin immunofluorescence staining in PANC-1^PAR^ and monoclonal cells. F-actin (red), DAPI (blue) (×60, scale bar = 20 μm). *G*: E-Cadherin protein levels were examined by western blot, intensities of immunoblots were quantified and normalized with GAPDH (*n* = 3) by ImageJ software. *H*: E-Cadherin mRNA levels were measured at the passage of 3, 5, and 10 in PANC-1^PAR^ and monoclonals by qPCR (*n* = 3). Data are represented as means ± SD. Immunoblotting and qPCR analyses were repeated independently three times. One-way ANOVA followed by Duncan’s test. *P < 0.05, **P < 0.01, ***P < 0.001, ****P < 0.0001.

We obtained 22 single-cell subclones from PANC-1 by limited dilution (Fig. 1*A*), followed by the initial assessment of c-FOXP3 expression through Western blot (data not shown). Then we choose four subclones to analyze the molecular and functional aspects according to c-FOXP3 expression level. Two cells with high expression of c-FOXP3 and two with low (Fig. 1*B*). To prevent artifact staining, we utilized quantitative real-time polymerase chain reaction (qPCR) to confirm the expression of c-FOXP3. We performed random testing of cells within 10 passages, discovering clone P2G8 that consistently exhibited high and stable c-FOXP3 expression (Fig. 1*C*).

These four subclones exhibited significant differences in morphology when cultured, setting them apart from the original PANC-1 cells (termed as PANC-1^PAR^) (Fig. 1*D*). Clone P2G8 (c-FOXP3^+^) appeared predominantly spherical and smaller in size, resembling undifferentiated cells with a loosely attached morphology. In contrast, clone P1G8 (c-FOXP3^−^) exhibited a spindle-shaped morphology, while clone P2D6 (c-FOXP3^+^) and P1E6 (c-FOXP3^−^) retained an epithelial-like morphology with tightly packed paving stone-like aggregates. We then performed crystalline violet staining to visualize cell morphologies, and F-actin immunofluorescence staining to analyze the cytoskeleton (Fig. 1, *E* and *F*). Fluorescence microscopy revealed distinct patterns of F-actin fibers in different clones. Notably, clone P2G8 exhibited reduced F-actin fibers, while the remaining cells showed consistent and intense F-actin fiber staining throughout the cytoplasm (Fig. 1*F*).

The differences in cell morphology led us to investigate the expression of E-Cadherin, a protein associated with cell-cell adhesion. Indeed, our Western blot analysis confirmed that clone P2D6 (c-FOXP3^+^E-Cadherin^+^) and P1E6 (c-FOXP3^−^E-Cadherin^+^) with epithelial-like morphology exhibited high expression levels of E-Cadherin, whereas clone P2G8 (c-FOXP3^+^E-Cadherin^−^) and P1G8 (c-FOXP3^−^E-Cadherin^−^) showed minimal expression (Fig. 1*G*). We further validated these findings by qPCR, indicating that E-Cadherin maintained relatively stable expression levels (Fig. 1*H*).

Based on these collective findings, we plan to explore cells that co-express both c-FOXP3 and E-Cadherin to further investigate their potential significance.

### Whole Transcriptome and Weighted Gene Co-Expression Network Analysis

**Figure 2.**
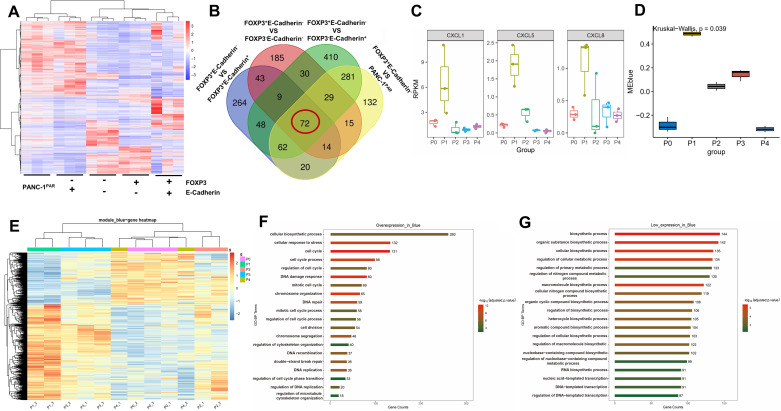
Differential gene and pathway analysis. *A*: heatmap of difference genes in PANC-1^PAR^and monoclonals. *B*: Venn diagram analysis shows the number of transcripts that are up-regulated in FOXP3^+^E-Cadherin^−^ cells compared with PANC-1^PAR^ and other monoclonals. *C*: box plot for CXCL1, CXCL5, and CXCL8 in PANC-1^PAR^ and monoclonals. *D*: box plot for differential gene in bule module in PANC-1^PAR^ and monoclonals. *E*: heatmap of differential gene expression in blue module. *F*: upregulated pathways in GO enrichment analysis. *G*: downregulated pathways in GO enrichment analysis. P0: PANC-1^PAR^, P1: P2G8 (c-FOXP3^+^E-Cadherin^−^ cells), P2: P2D6 (c-FOXP3^−^E-Cadherin^-^ cells), P3: P1G8 (c-FOXP3^+^E-Cadherin^+^ cells), P4: P1E6 (c-FOXP3^−^E-Cadherin^+^ cells).

We conducted whole transcriptome sequencing on PANC-1^PAR^ and four monoclonal cells and discovered that their molecular expression varied (Fig. 2*A*). We identified 72 genes that were significantly upregulated in clone P2G8 (c-FOXP3^+^E-Cadherin^−^) compared with the other groups by using Venn diagram analysis (Fig. 2*B*). The chemokines, including *C-X-C motif ligand 1 (CXCL1)*, *C-X-C motif ligand 5 (CXCL5)*, and *C-X-C motif ligand 8 (CXCL8)* were found to be significantly elevated in c-FOXP3^+^E-Cadherin^−^ cells (Fig. 2*C*).

**Figure 3.**
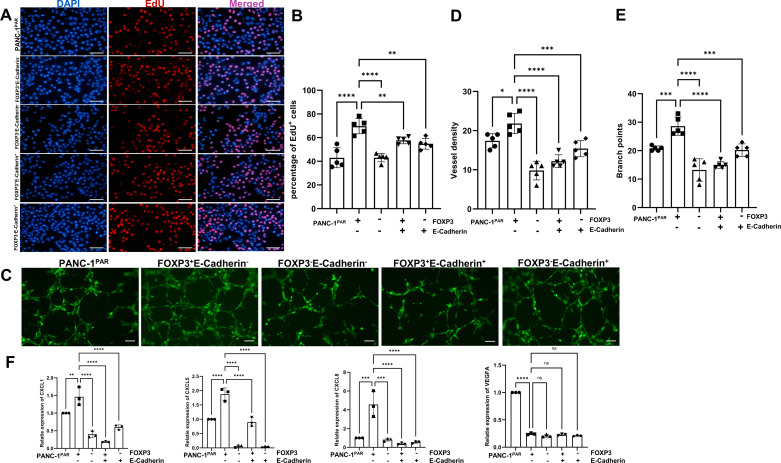
Detection of monoclonal cell proliferation and pro-angiogenesis ability. *A*: EdU assay (*n* = 5) (×20, scale bar = 100 μm). *B*: quantitation of percentage of EdU^+^ cells, EdU^+^ cells were quantified by ImageJ software (Image Processing and Analysis in Java; National Institutes of Health; http://imagej.nih.gov/); *n* = 5. *C*: tube formation assays (*n* = 5) (×10, scale bar = 100 μm). *D*: quantitation of vessel density in the subclones; *n* = 5. *E*: quantitation of branch points in the subclones; *n* = 5. *F*: qPCR examine the CXCL1, CXCL5, CXCL8, and VEGFA expression (*n* = 3). Data are represented as means ± SD. Statistical significance was calculated by one-way ANOVA followed by Duncan’s test. *P < 0.05, **P < 0.01, ***P < 0.001, ****P < 0.0001.

To gain further insights into the biological functions of c-FOXP3^+^E-Cadherin^−^ cells, we performed WGCNA analysis. First, we used transcriptome data from 15 cell samples to cluster and exclude the obviously aberrant samples by setting a threshold (Supplemental Fig. S1*A*). We then set the soft threshold to 10 (Supplemental Fig. S1*B*) and visualized the primed and merged modules under the clustering tree (Supplemental Fig. S1*C*). The reliability of module delineation was supported by transcription correlation analysis, which revealed no significant linkage between modules (Supplemental Fig. S1*E*). Moreover, the correlation analysis between modules showed no substantial associations (Supplemental Fig. S1*F*). Next, we examined the frontal correlations between the ME (module eigengene) values and cell clones to investigate the relationship between modules and cell functions. The green and blue modules were found to be positively correlated with c-FOXP3^+^E-Cadherin^−^ cells (Supplemental Fig. S1*D*, Fig. 3, *A* and *B*, Fig. 2, *D* and *E*). To gain further insights into the biological functions of the differentially expressed genes (DEGs) in these modules, we performed Gene Ontology (GO) enrichment analysis. The blue modules were found to be mainly linked with the cell cycle and DNA replication (Fig. 2*E*), while the green modules showed associations with cellular biosynthetic processes (Supplemental Fig. S2, *A–D*).

Taken together, the upregulation of specific chemokines and the enrichment of cell cycle in c-FOXP3^+^E-Cadherin^−^ cells may shed light on its unique characteristics and functional properties.

### C-FOXP3^+^E-Cadherin^−^ Cells Contribute to Cell Proliferation and Angiogenesis in Vitro

Based on the previous analysis, we selected key functions to investigate further, focusing on cell proliferation and angiogenesis. The c-FOXP3^+^E-Cadherin^−^ cells exhibited higher rates of proliferation in vitro by the EdU assay (Fig. 3, *A* and *B*). The incorporation of EdU in DNA increased, indicating that c-FOXP3^+^E-Cadherin^−^ cells may promote cell proliferation by regulating DNA synthesis and cell cycle progression. We further conducted a pro-angiogenic assay, culturing human umbilical vein endothelial cells (HUVECs) in a conditioned medium from PANC-1^PAR^ and monoclonal cells. C-FOXP3^+^E-Cadherin^−^ cells demonstrated a significant pro-angiogenic capacity (Fig. 3, *C–E*).

Furthermore, we utilized qPCR to identify specific genes involved in angiogenesis (Fig. 2*C*). In c-FOXP3^+^E-Cadherin^−^ cells, CXCL1, CXCL5, and CXCL8 showed substantial upregulation, but vascular endothelial growth factor A (VEGFA) was not (Fig. 3*F*). These findings suggest that c-FOXP3^+^E-Cadherin^−^ cells promotes angiogenesis, potentially through the secretion of CXCL1, CXCL5, and CXCL8, rather than relying on VEGFA.

Our experimental evidence indicates that c-FOXP3^+^E-Cadherin^−^ cells are more proliferative and possess a potent pro-angiogenic capacity.

### C-FOXP3^+^ Cells Contribute to Cell Proliferation and Angiogenesis in Vivo

**Figure 4.**
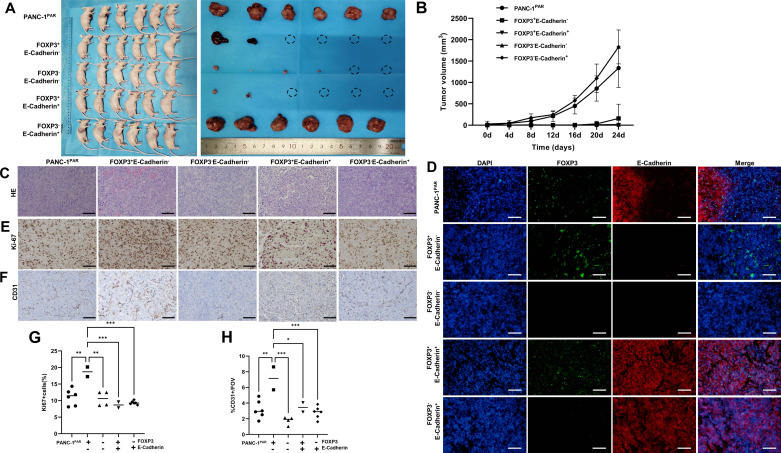
Subcutaneous xenograft. *A*: subcutaneous tumor models (*n* = 6 per groups). *B*: tumor volumes were recorded every 4 days. *C*: H&E of the tumor. *D*: C-FOXP3 and E-Cadherin were detected in subcutaneous tumors by immunofluorescence co-staining. C-FOXP3 (green), E-Cadherin (red) and DAPI (blue). *E*: Ki-67 index was examined by IHC in each group. *F*: CD31 was examined by IHC in each group (*C*–*F*, ×20, scale bar = 100 μm). *G*, *H*: Ki-67 index and CD31 count in each group were quantified by ImageJ software (Image Processing and Analysis in Java; National Institutes of Health; http://imagej.nih.gov/). Data are presented as the median of each group and analyzed using one-way ANOVA (and nonparametric or mixed) followed by Tukey’s test. *P < 0.05, **P < 0.01, ***P < 0.001.

To clarify the in vivo role of the monoclonal cells, we performed further investigations using subcutaneous tumor mouse model (Fig. 4*A*). Notably, we observed that when the volumes of tumors in the PANC-1^PAR^ and c-FOXP3^−^E-Cadherin^+^ cells had reached 1 cm^3^, the other groups exhibited smaller size (Fig. 4, *A* and *B*). After dissection, we examined the subcutaneous tumor tissue, using hematoxylin and eosin (H&E) staining to reveal the distinct morphology of the monoclonal cells (Fig. 4*C*). Subsequently, c-FOXP3 and E-Cadherin were detected in subcutaneous tumors by immunofluorescence double labeling. The results indicated that the molecular expression pattern of the cells remained consistent with the in vitro observations (Fig. 4*D*).

To validate the proliferative activity of these tumor cells and their impact on angiogenesis, we detected the Ki-67 proliferation index and CD31 by immunohistochemistry. Despite the c-FOXP3^+^E-Cadherin^−^ group tumor volume being small, the Ki-67 proliferation index was high, indicating active cell proliferation (Fig. 4, *E* and *G*). Moreover, the appearance of c-FOXP3^+^E-Cadherin^−^ cells-formed tumors appeared redder than those of other groups, and the CD31 level was also elevated, suggesting an increased presence of blood vessels, which could account for the observed appearance (Fig. 4, *F* and *H*).

Collectively, we conclude that these subclones not only maintain stable molecular expression but also retain consistent functionality both in vivo and in vitro.

### C-FOXP3^+^E-Cadherin^−^ Cells in Pancreatic Cancer Are Related with Poor Prognosis

**Figure 5.**
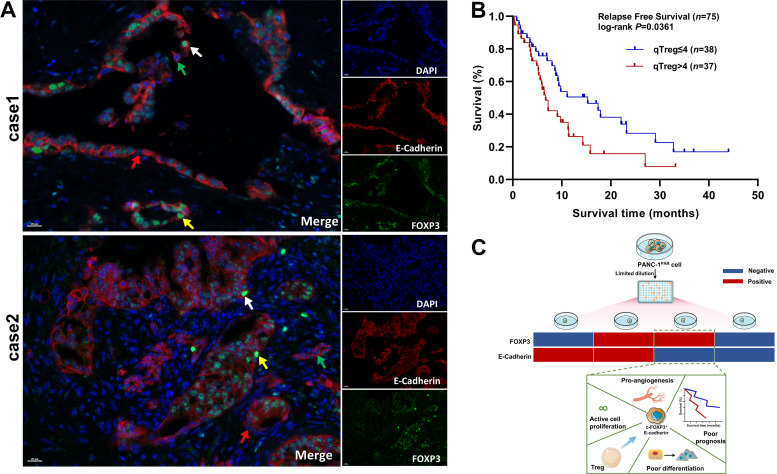
C-FOXP3^+^E-Cadherin^−^ cells in pancreatic ductal adenocarcinoma (PDAC) are related with poor prognosis. *A*: representative pictures of immunofluorescence analysis for c-FOXP3^+^E-Cadherin^−^ (yellow arrows), c-FOXP3^+^E-Cadherin^+^ (white arrows), c-FOXP3^−^E-Cadherin^+^ (green arrows) and c-FOXP3^−^E-Cadherin^−^ (red arrows) cells in PDAC, field of view (FOV). C-FOXP3 (green), E-Cadherin (red), and DAPI (blue) (×40, scale bar = 20 μm). *B*: Kaplan–Meier curves for relapse free survival (RFS) in a cohort of patients (*n *= 75) diagnosed with PDAC, according to c-FOXP3^+^E-cadherin^−^ cells count. *P* values determined by log-rank Mantel–Cox test. *C*: the characteristics of c-FOXP3^+^E-cadherin^−^ cells.

To investigate the presence of cells resembling c-FOXP3^+^E-Cadherin^−^ cells in human PDAC, we collected 75 tissue samples from patients who underwent radical surgery. C-FOXP3 and E-Cadherin in human PDAC tissues were identified through immunofluorescence co-staining. The results revealed that these four subpopulations, including FOXP3^+^E-cadherin^+^, FOXP3^+^E-cadherin^−^, FOXP3^−^E-cadherin^+^, and FOXP3^−^E-cadherin^−^ cells, were also existed in PDAC tissue sample (Fig. 5*A*).

We counted the average number of c-FOXP3^+^E-Cadherin^−^ tumor epithelial cells in five randomly selected fields at ×20 magnification per slide. The high c-FOXP3^+^E-Cadherin^−^ cells group (high c-FOXP3^+^E-Cadherin^−^ cells group >4 cells/high-power field and low group ≤4 cells/high-power field) correlated with unfavorable clinicopathological parameters, including poorer tumor differentiation, lymph node metastasis, vascular invasion, and the count of Tregs (all *P* < 0.001) (Table 1). However, we did not observe a significant correlation between c-FOXP3^+^E-Cadherin^−^ cells and the T stage (*P* = 0.399), suggesting that this cell subtype may not affect the tumor volumes. We conducted relapse-free survival (RFS) analysis using the Kaplan-Meier curve (median time, 6.7 and 15.3 mo, *P* = 0.0361, Fig. 5*B*), suggesting that c-FOXP3^+^ cells are a potential biomarker for poor prognosis of PDAC.

**Table 1.** The relationship between the clinicopathological factors and the c-FOXP3^+^E-Cadherin^−^ cell count

Clinicopathological Factors	c-FOXP3^+^E-Cadherin^−^ Cell (Numbers)		χ^2^	*P* Value
	≤4	>4		
Gender				
Male	23	22	0.009	0.925
Female	15	15		
Age, yr				
≤55	8	8	0.004	0.952
>55	30	29		
Differentiation				
Good/moderate	32	15	15.281	0.000***
Poor	6	22		
T stage				
T1–T2	29	25	0.712	0.399
T3	9	12		
Nodal status				
N0	29	15	9.895	0.002**
N1–N2	9	22		
Nerve invasion				
Negative	12	5	3.490	0.062
Positive	26	32		
Vascular invasion				
Negative	33	22	7.188	0.007**
Positive	5	15		
Treg (numbers)				
<15	33	15	17.443	0.000***
≥15	5	22		

**P < 0.01, ***P < 0.001 (*P* values were calculated using Pearson’s chi-square test, *n* = 75).

These data suggest that c-FOXP3^+^E-Cadherin^−^ cells may play a significant role in PDAC progression and clinical outcomes, making them potential targets for further research and therapeutic intervention.

## DISCUSSION

In this report, we identified an epithelial subset with stable c-FOXP3 expression. These cells exhibit robust proliferation and angiogenic capabilities. Our findings expand traditional epithelial cell definitions and introduce lymphocyte markers to these subpopulations, offering novel avenues for targeted tumor therapy and angiogenesis intervention.

We observed a significant enrichment of pathways related to cell cycle and DNA replication in c-FOXP3^ + ^E-Cadherin^−^ cells through transcriptome sequencing and WGCNA analysis. Previous studies have indicated that increased FOXP3 expression leads to a higher proportion of cells in a resting phase (G0/G1 phase), reduces their progression into the DNA replication phase (S phase), and also enhances cell apoptosis(20). C-FOXP3^+^E-Cadherin^−^ cells formed smaller tumors but exhibited an increased Ki-67 index, consistent with EdU assay in vivo. This underscores a dichotomy between differentiation and proliferative potential, with these cells showing heightened in vitro proliferation, potentially due to an undifferentiated or stem cell state(21). Diverse signals can trigger cell differentiation or morphological changes in vivo, as seen in the immunofluorescence staining results showing the loss of c-FOXP3^+^ cells in parts of the tumor tissue formed by c-FOXP3^+^E-Cadherin^−^ cells. Such differentiation could prioritize processes like angiogenesis over growth, resulting in smaller tumors. Cell cycle distribution disparities further contribute, with EdU marking S-phase cells and Ki-67 spanning S to M phases(22, 23). In vivo tumor formation might involve altered cell cycle patterns, elevating proliferation without increasing volume. Balancing factors, like apoptosis, could further restrain growth. Finally, the microenvironment, including oxygen, nutrients, and cell interactions, undoubtedly modulates cell behavior, impacting tumor size.

Vascular endothelial growth factor A (VEGFA) is a key driver of angiogenesis and a prime therapeutic target (24). Tumor cells and stromal-derived VEGFA prompts endothelial cell growth, new blood vessel formation, and tumor progression. The conventional approach of using anti-VEGF agents, often in conjunction with chemotherapy or immunotherapy, has been established as the standard therapeutic strategy across various malignancies(25). The benefits of anti-VEGF treatment have been firmly established in colorectal cancer and have extended to various other tumor types, such as non-squamous non-small cell lung cancer, glioblastoma multiforme, and ovarian cancer(26–29). Previous studies showed that c-FOXP3 inhibits VEGF transcription directly by binding to its promoter via specific forkhead-binding motifs, consequently diminishing angiogenesis-inducing potential in HUVECs and downregulating VEGF expression in breast cancer cells (30). We demonstrated that c-FOXP3^+^E-Cadherin^−^ cells promoted the tube-forming ability of HUVECs, even with low VEGFA expression, indicating a VEGFA-independent angiogenic mechanism. Transcriptome analysis and mRNA detection revealed elevated CXCL1, CXCL5, and CXCL8 levels in c-FOXP3^+^E-Cadherin^−^ cells. CXCL1, CXCL5, and CXCL8 are chemokines that can bind to C-X-C chemokine receptor 1 (CXCR1) and C-X-C chemokine receptor 2 (CXCR2) to activate intracellular signaling cascades and promote angiogenesis (31–34). These chemokines also play a pivotal role in recruiting immune cells and endothelial precursor cells, which are involved in angiogenesis (35). C-FOXP3^+^E-Cadherin^−^ tumor epithelial cells can rejuvenate tumor angiogenesis in a VEGF-independent manner, elucidating potential resistance mechanisms to anti-VEGF therapy in patients with PDAC. Consequently, strategies aimed at modulating the activity of CXCL1, CXCL5, and CXCL8 hold promise as potential therapeutic interventions to impede tumor angiogenesis.

In this investigation, we unveiled a noteworthy correlation between a higher count of c-FOXP3^+^E-Cadherin^−^ cells and poorer tumor differentiation, lymph node metastasis, vascular invasion, and unfavorable prognosis in PDAC (Fig. 5*C*). Moreover, the quantity of c-FOXP3^+^E-Cadherin^−^ cells does not relate to tumor T stage but links to a lower degree of differentiation. This hints at c-FOXP3^+^E-Cadherin^−^ cells potentially being a key subgroup in early tumorigenesis, with some cells losing the molecular and functional characteristics of c-FOXP3^+^ cells as they differentiate. This finding holds crucial implications for guiding clinical strategies. The number of c-FOXP3^+^E-Cadherin^−^ epithelial cells correlated with Tregs. It implied that c-FOXP3^+^E-Cadherin^−^ may be an important component of the tumor microenvironment. In PDAC tissue, the high number of c-FOXP3^+^E-Cadherin^−^ cells was associated with relapse-free survival (RFS). It implies that patients with higher counts of these cells might have a differential risk of disease relapse after treatment. Clinically, this could influence decisions regarding treatment intensities, follow-up monitoring frequency, and patient counseling about their potential disease trajectory. A phase II trial in locally advanced pancreatic cancer patients revealed elevated c-FOXP3^+^ cell counts in non-responders who received losartan alongside FOLFIRINOX (FFX) and chemoradiation (36). This suggests that the number of c-FOXP3^+^ cells could serve as a marker to assess therapeutic efficacy in pancreatic cancer.

This study elucidated the intrinsic molecular and functional characteristics of naturally occurring c-FOXP3^+^ epithelial cells (Fig. 5*C*). However, several potential limitations of this study should be noted. The study primarily centered on the PANC-1 cell line, which might not fully represent the complexity of various tumor microenvironments. We have successfully generated genetically engineered mice with epithelial-specific FOXP3 knock-in, which will enable further investigation into the molecular and functional characteristics of these cells. Furthermore, the mechanisms underlying the VEGF-independent angiogenic promotion by c-FOXP3^+^ epithelial cells need further exploration. We will use inhibitors to block chemokines and their receptors to determine whether CXCL1, CXCL5, and CXCL8 act independently or synergistically to promote angiogenesis. Our current investigation into the characteristics of c-FOXP3^+^ cells represents pioneering research. Going forward, we intend to address existing research limitations and elucidate the molecular mechanisms governing the preservation of this cell phenotype, as well as its implications for cellular fate.

In conclusion, we identified an epithelial cell subset that consistently expresses the traditionally lymphocyte-associated FOXP3. Their expression in tumors can predict clinical outcomes and gauge anti-vascular treatment efficacy. Our research challenges conventional views on epithelial cell definitions and broadens FOXP3’s designation beyond just an immune cell marker.

## DATA AVAILABILITY

Data will be made available upon reasonable request.

## SUPPLEMENTAL DATA

10.6084/m9.figshare.24550735 Supplemental Figs. S1 and S2: https://doi.org/10.6084/m9.figshare.24550735.

## GRANTS

This study was supported by funding from the National Natural Science Foundation of China: 82125026, 82330081 (to H.R.) and 82303933 (to Q.Y.); Taishan Scholars Program of Shandong Province: Ts20190987(to H.R.); Major State Basic Research Development Program of Natural Science Foundation of Shandong Province in China: ZR2020ZD11 (to H.R.); Shandong Province Postdoctoral Innovation Talent Program: SDBX2022022 (to Y.Z.).

## DISCLOSURES

No conflicts of interest, financial or otherwise, are declared by the authors.

## AUTHOR CONTRIBUTIONS

H.R. conceived and designed research; R.G., X.C., and X.S. performed experiments; Y.Z. analyzed data; R.G. interpreted results of experiments; R.G. prepared figures; R.G. drafted manuscript; J.W., Q.Y., and K.L. edited and revised manuscript; H.R. approved final version of manuscript.
